# Differential Associations between *CDH13* Genotypes, Adiponectin Levels, and Circulating Levels of Cellular Adhesive Molecules

**DOI:** 10.1155/2015/635751

**Published:** 2015-10-27

**Authors:** Ming-Sheng Teng, Semon Wu, Lung-An Hsu, Hsin-Hua Chou, Yu-Lin Ko

**Affiliations:** ^1^Department of Research, Taipei Tzu Chi Hospital, Buddhist Tzu Chi Medical Foundation, New Taipei City 231, Taiwan; ^2^Department of Life Science, Chinese Culture University, Taipei 111, Taiwan; ^3^The First Cardiovascular Division, Department of Internal Medicine, Chang Gung Memorial Hospital and Chang Gung University College of Medicine, Taoyuan 333, Taiwan; ^4^The Division of Cardiology, Department of Internal Medicine and Cardiovascular Center, Taipei Tzu Chi Hospital, Buddhist Tzu Chi Medical Foundation, New Taipei City 231, Taiwan; ^5^School of Medicine, Tzu Chi University, Hualien 970, Taiwan

## Abstract

*CDH13* gene variants with lower adiponectin levels are paradoxically associated with a more favorable metabolic profile. We investigated the statistical association between *CDH13* locus variants and adiponectin levels by examining 12 circulating inflammation marker levels and adiposity status in 530 Han Chinese people in Taiwan. After adjustments for clinical covariates, adiponectin levels were positively associated with soluble vascular cell adhesion molecule-1 (sVCAM1) levels and negatively associated with adiposity status and levels of C-reactive protein (CRP), soluble E-selectin (sE-selectin), and soluble intercellular adhesion molecule-1 (sICAM1). In addition, minor alleles of the *CDH13* rs12051272 polymorphism were found to have lower adiponectin levels and higher CRP, sE-selectin, sICAM1, and sVCAM1 levels as well as higher body mass indices and waist circumferences in participants (all *P* < 0.05). In a subgroup analysis stratified by sex, significant associations between *CDH13* genotypes and sE-selectin levels occurred only in men (*P* = 3.9 × 10^−4^ and interaction *P* = 0.005). *CDH13* locus variants and adiponectin levels are associated with circulating levels of cellular adhesion molecules and adiposity status in a differential manner that interacts with sex. These results provide further evidence for the crucial role of adiponectin levels and *CDH13* gene variants in immune-mediated and inflammatory diseases.

## 1. Introduction

Adiponectin, a multimeric protein and one of the most abundant gene products expressed in adipose tissue [1], is well known to play a critical role in metabolic regulation, affecting obesity, insulin sensitivity, and atherosclerosis [2]. Several studies have shown that adiponectin is involved in numerous biological effects, including antidiabetic, antioxidant, and antiatherosclerotic actions [3]. Circulating levels of adiponectin are reduced in patients with obesity and associated comorbidities [4], and inflammation is crucial in downregulating adiponectin production [5]. By contrast, elevated systemic and local levels of adiponectin are present in patients with immune-mediated and inflammatory diseases [6]. Whereas previous studies have demonstrated differential associations between circulating adiponectin and inflammatory marker levels [7–12], conflicting data have been reported regarding the proinflammatory and anti-inflammatory effects of adiponectin in* in vitro* and* in vivo* studies [13]. These results suggested a multifaceted influence of adiponectin in inflammation, occurring through various mechanisms involved in modifying circulating adiponectin levels and in regulating downstream adiponectin-related signal pathways [13].

T-cadherin, of the cadherin superfamily of the transmembrane proteins that mediate calcium-dependent intercellular adhesion, is the receptor for hexameric and high-molecular weight adiponectin expressed in the vasculature [14] and cardiac myocytes [15]. The* CDH13* gene, which encodes T-cadherin, is localized at chromosome 16q23.3, spans 1.2 Mb, and contains 14 exons. Binding a low-density lipoprotein or adiponectin to T-cadherin can activate an NF*κ*B signaling pathway, which plays a central role in inflammation and links obesity and vascular disease [16]. Genome-wide association studies (GWAS) have shown associations between* CDH13* genotypes and haplotypes with adiponectin levels [17–19]. A meta-analysis reported the* CDH13* gene region to be the most crucial locus associated with adiponectin levels [20]. Paradoxically, genotypes with lower adiponectin levels have more favorable metabolic phenotypes. After mediation analysis, our data showed the* CDH13* rs12051272 polymorphism to be the most significant* CDH13* variant associated with metabolic phenotypes and metabolic syndrome in Han Chinese people in Taiwan [21]. Although T-cadherin has been associated with immune-mediated diseases [22], the role of* CDH13* variants in inflammatory marker levels has not been previously investigated. The current study elucidates the role of* CDH13* genotypes and adiponectin levels in inflammatory marker levels, which affect various stages of atherosclerosis progression. The interactive effects of sex and obesity on the genotype-phenotype associations were also analyzed.

## 2. Subjects and Methods

### 2.1. Study Population

This study was approved by the institutional review board of Taipei Tzu Chi Hospital, Buddhist Tzu Chi Medical Foundation (IRB number: 02-XD56-120). The study population was previously reported [23]. The exclusion criteria included cancer, current renal or liver disease, and a history of myocardial infarction, stroke, or transient ischemic attacks. In brief, 617 Han Chinese subjects were recruited during routine health examinations between October 2003 and September 2005 at Chang Gung Memorial Hospital. All participants provided written, informed consent. Participants answered a questionnaire on their medical history and lifestyle characteristics and underwent a physical examination that involved measurements of height, weight, waist circumference, and blood pressure (BP) in a sitting position after 15 min of rest. Fasting blood samples were obtained from each participant. Obesity was defined as body mass index (BMI) ≥ 25 kg/m^2^ according to the Asian criteria [24]. Current smokers were defined as those who smoked cigarettes regularly at the time of survey. Participants aged < 18 years or with a history of regular use of medications for diabetes mellitus, hypertension, and/or lipid-lowering drugs were excluded from the analysis. Participants with hypertension, defined as a systolic BP ≥ 140 mm Hg, a diastolic BP ≥ 90 mm Hg, or both, and not taking antihypertensive drugs and those with diabetes mellitus, defined as blood sugar levels before a meal of ≥7.0 mmol/L, and not taking medications for diabetes mellitus were included for analysis. In all, 530 study participants were enrolled for analysis (mean ± SD): 270 men, age = 43.9 ± 9.3 years, and 260 women, age = 45.9 ± 9.3 years.  Table 1 summarizes the clinical and biometric features of the study group.

### 2.2. Genomic DNA Extraction and Genotyping

Genomic DNA was extracted as reported previously [25]. The* CDH13* rs12051272 polymorphism that was previously reported to be strongly associated with adiponectin levels and metabolic syndrome [21] was chosen in this study. Genotyping was performed using TaqMan SNP Genotyping Assays from Applied Biosystems (ABI, Foster City, CA, USA).

### 2.3. Laboratory Examinations and Assays

Before starting the study, all participants underwent an initial screening assessment that included a medical history and novel risk factors. A total of 15 mL of venous blood was collected in the morning after an overnight (8–12 h) fast. Venous blood samples including serum and plasma were collected from an antecubital vein and centrifuged at 3000 ×g for 15 min at 4°C. Immediately thereafter, serum and plasma samples were frozen and stored at −80°C prior to analysis. Plasma fibrinogen levels were measured in a central laboratory as previously reported [23]. Most markers, including serum adiponectin, C-reactive protein (CRP), serum amyloid A (SAA), soluble intercellular adhesion molecule-1 (sICAM1), soluble vascular cell adhesion molecule-1 (sVCAM1), soluble E-selectin (sE-selectin), matrix metalloproteinase-9 (MMP9), and plasma monocyte chemotactic protein-1 (MCP1), were measured using an enzyme-linked immunosorbent assay (ELISA) developed in-house. Measurement of sICAM1 was performed using the R&D System ICAM1 assay monoclonal antibody. All in-house kits exhibited strong correlation compared with commercially available ELISA kits [23]. Circulating serum matrix metalloproteinase-2 (MMP2) and plasma matrix metalloproteinase-1 (MMP1), soluble P-selectin (sP-selectin), soluble tumor necrosis factor receptor 2 (sTNFRII), and interleukin-6 (IL6) were measured using commercially available ELISA kits from R&D (Minneapolis, MN, USA).

### 2.4. Statistical Analysis

The chi-square test was used for testing to compare categorical variables of diabetes mellitus and smoking. The clinical characteristics that were continuous variables are expressed as means ± SDs and were tested using a two-sided *t*-test or analysis of variance (ANOVA). Pearson correlation coefficients (*r*) were calculated to determine the association between adiponectin levels and clinical and biochemical factors with the adjustment of age and sex. Furthermore, a general linear model was applied to capture the major effect of each polymorphism on clinical and biochemical variables, with BMI, age, gender, and smoking status as confounding covariates. We also used dominant models for numeric association test after recoding our SNPs from categorical variables to continuous variables, such as 0, 1 of a particular allele. A value of *P* < 0.05 using two-sided tests was considered statistically significant. All the above calculations were performed with standard statistical SPSS 12 software (SPSS, Chicago, IL, USA). All of the biomarker levels, besides MMP9, were logarithmically transformed before statistical analysis to adhere to a normality assumption. In addition, stepwise linear regression analysis was used to analyze independent predictors of adiponectin levels. We further analyzed the influence of interaction between* CDH13* rs12051272 genotypes with obesity and gender on inflammatory marker levels.

## 3. Results

### 3.1. Associations between Circulating Adiponectin and Inflammatory Marker Levels

Table 1 summarizes the demographic features, clinical profiles, and biomarker levels of the study participants. The associations between adiponectin and inflammatory marker levels are shown in Table 2. Elevated adiponectin levels were found with increasing age. After adjustments for clinical covariates, a significant positive correlation was observed between adiponectin and sVCAM1 levels, and significant negative correlations were observed between adiponectin levels and circulating levels of various inflammatory biomarkers, including CRP, sE-selectin, and sICAM1, and with adiposity status, including body mass index (BMI) and waist circumference (Table 2).

### 3.2. Multivariate Analysis

Stepwise linear regression analysis in a model including age, sex, BMI, waist circumference, smoking status, and various inflammatory marker levels revealed that being female (*P* = 7.9 × 10^−13^), sVCAM1 (*P* = 7.49 × 10^−7^), and age (per year; *P* = 0.001) were positively associated with adiponectin levels, whereas BMI (*P* = 1.71 × 10^−6^) and circulating levels of sE-selectin (*P* = 0.003), sICAM1 (*P* = 0.003), and CRP (*P* = 0.002) were negatively associated with lower adiponectin levels (Table 3). When* CDH13 rs12051272* genotypes were further enrolled in multivariate analysis, all associations remained significant (data not shown).

### 3.3. Associations between* CDH13* Gene Variant rs12051272 and Inflammatory Marker Levels

Table 4 shows the associations of* CDH13* rs12051272 genotypes with adiponectin, inflammatory marker levels, and adiposity status. The T-allele of rs5491 was demonstrated to alter a critical binding site of ICAM1, interfering with the monoclonal antibody used in the R&D System ICAM1 assay for binding the protein [26]. To study the associations of sICAM1 levels, we enrolled only subjects with the* ICAM1* rs5491 AA genotype for analysis. After adjustments for age and sex, the* CDH13* genotypes were found to be positively associated with CRP, sE-selectin, sICAM1, and sVCAM1 levels and with BMI and waist circumference. With an additional adjustment for BMI, the association remained in circulating levels of sE-selectin, sICAM1, and sVCAM1 (*P* = 0.039, *P* = 0.037, and *P* = 0.010, resp.), whereas a trend of higher sP-selectin level (*P* = 0.064) was also noted. After further adjustment for adiponectin levels, the association between* CDH13* rs12051272 genotypes and sVCAM1 levels became more significant (*P* = 0.001).

### 3.4. Subgroup and Interaction Analyses by Gender

**Figure 1:**
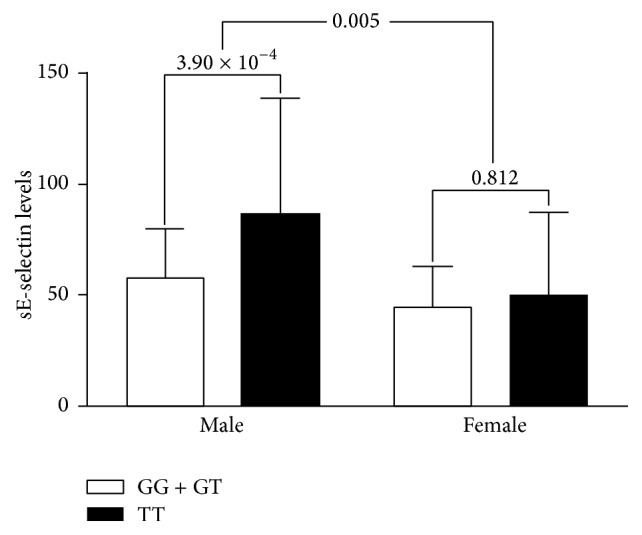
Comparison of levels of soluble E-selectin according to* CDH13* rs12051272 genotypes in the recessive model between Han Chinese men and women in Taiwan. After adjustments for clinical covariates, minor alleles of* CDH13* rs12051272 genotypes were found to be associated with higher sE-selectin levels, predominantly in men (*P* = 3.90 × 10^−4^). Interaction analysis revealed an interaction of sex with the* CDH13* rs12051272 genotypes (interaction *P* = 0.005).

As presented in Figure 1, after adjustments for clinical covariates, subgroup and interaction analyses revealed an association of* CDH13* rs12051272 genotypes with sE-selectin levels only in men (*P* = 3.9 × 10^−4^, interaction *P* = 0.005). There was no evidence of interaction by obesity or sex in the association between rs12051272 genotypes and other inflammatory biomarker levels.

## 4. Discussion

This study analyzed the association of* CDH13* locus variant rs12051272 and adiponectin levels with inflammation marker levels and adiposity status in Han Chinese subjects in Taiwan. Our data revealed that circulating cellular adhesion molecule levels were associated with adiponectin levels and* CDH13* gene variants in a differential manner. Subgroup analysis demonstrated different genetic backgrounds by gender in the association between* CDH13* variants and sE-selectin levels. These results provide further evidence that T-cadherin is the target adiponectin receptor for inflammation and that adiponectin levels and* CDH13* gene variants are crucial in immune-mediated and inflammatory diseases.

### 4.1. Association between Circulating Adiponectin and Inflammatory Marker Levels

Ouchi et al. first demonstrated that physiological concentrations of adiponectin exert significant inhibitory effects on TNF*α*-induced monocyte adhesion and adhesion molecule expression in a dose-dependent manner. Adiponectin specifically suppressed TNF*α*-induced I*κ*B-NF*κ*B activation through a cAMP-dependent pathway in human arterial endothelial cells [8]. Similar to our results, consistent negative correlations have been reported between adiponectin levels and circulating CRP and sE-selectin levels in healthy and diseased populations [9, 12, 27–30]. Our data indicate a negative association between adiponectin levels and sICAM1 levels that had not been previously reported. Another interesting finding consistent with the literature is that sVCAM1 is the only inflammatory marker level positively associated with adiponectin levels in our study and in published articles [10, 11]. Although the exact mechanism of this association has not been fully elucidated, Vaverkova et al. hypothesized that adiponectin may be involved in the shedding of the ectodomain of VCAM1 from the endothelial surface, thereby increasing the serum level of VCAM1 [10].

### 4.2. Association of* CDH13* Variants with Inflammatory Marker Levels

This study is the first to demonstrate that* CDH13* genotypes are associated with inflammatory marker levels. The association occurred predominantly in circulating levels of cellular adhesion molecules, which may be dependent on or independent of adiponectin levels. Three adiponectin binding receptors have been cloned: adiponectin R1, adiponectin R2, and T-cadherin. Previous studies have suggested that nearly all of the metabolic effects of adiponectin are conferred by the adiponectin R1 and R2 receptors [16]. By contrast, the binding of adiponectin to T-cadherin can activate the NF*κ*B signaling pathway, which plays an essential role in inflammation and serves as a link between obesity and vascular disease [8]. T-cadherin was discovered to be a unique “truncated” cadherin that is associated with the plasma membrane but lacks cytoplasmic sequences and is not a classical receptor, which requires both ligand binding and intracellular signal capabilities [31]. T-cadherin sequesters adiponectin to the endothelium and heart and serves as an adiponectin repository; thus, in mice with T-cadherin deficiency, this adiponectin is accumulated in the circulation with elevated adiponectin levels [14, 15, 22]. However, in T-cadherin-deficient mice, some of adiponectin actions are not mediated because of lack of T-cadherin. Danzel et al. reported that adiponectin was unable to associate with cardiac tissue in T-cadherin-deficient mice, while physical association of adiponectin with T-cadherin was necessary for adiponectin-dependent AMP-activated protein kinase phosphorylation and adiponectin's physiological activity in the heart [15]. These data are consistent with a model in which T-cadherin serves the adiponectin binding, while yet-unknown transmembrane proteins, possibly including adiponectin R1 or R2, are required for transmitting the binding signal to intracellular signal pathways. Further, external application of adiponectin upregulates posttranscriptional expression of T-cadherin to enable docking of adiponectin to cardiomyocytes and endothelial cells and regulate tissue T-cadherin levels through a positive feedback loop that operates by suppressing phospholipase-mediated T-cadherin release from the cell surface [15, 32]. Thus, T-cadherin not only regulates circulating and tissue-bound adiponectin levels but also competes with the adiponectin R1 and R2 receptors for adiponectin binding and interferes with the coupling of both receptors to their downstream intracellular targets [32, 33]. The intracellular signaling events following the activation of adiponectin R1 and adiponectin R2 involve binding to the adaptor protein APPL1 (i.e., the adaptor protein containing pleckstrin homology domain) and then activating AMP-activated protein kinase, which may block the NF*κ*B pathways and downstream inflammation events [34]. Bag and Anbarasu analyzed functional gene interactions of the adiponectin gene and revealed that, in contrast to adiponectin and adiponectin R2 (which are involved mostly in glucose and lipid metabolic processes), the* CDH13* gene participates in the cell adhesion process with adiponectin and adiponectin R2 [35]. The above findings of complex interaction between adiponectin and T-cadherin may provide the base of our results showing that* CDH13* variants with lower T-cadherin expression may increase the adiponectin level and decrease cellular adhesive molecule levels. These findings further indicate that adiponectin acts through its receptors as an endogenous modulator of endothelial cell function via metabolic and inflammatory effects, which may be involved in endothelial dysfunction in earlier atherosclerotic processes [36].

### 4.3. Adiponectin Levels,* CDH13* Variants, and Adiposity Status

Obesity downregulates adiponectin levels through metabolic derangement and the dysregulation of inflammation [4]. Several studies have shown the association of* CDH13* variants with obesity or the syndrome of obesity [19, 37–39]. In a GWAS, Lee et al. identified the influence of gene-gene interaction between variants in* CDH13* and* SLC10A7* genes on the association with obesity [37]. Our data further demonstrated the association of the* CDH13* variant with BMI and waist circumference in Han Chinese subjects in Taiwan. The cause-effect relationship is currently unclear, and further study is required to determine its future clinical implications.

### 4.4. Effect of Sex on the Association between* CDH13* Variants and sE-Selectin Levels

The interplay between genetic and environmental factors is critical in the phenotype development of complex traits. We found that the association between* CDH13* genotypes and sE-selectin levels was predominant in men. Previous studies have shown gene-gene (epistatic effect) and gene-gender interactions between* CDH13* genotypes and metabolic syndrome and between* CDH13* genotypes and obesity, respectively [37, 40]. Estradiol and progesterone have been shown to be involved in the transcriptional and posttranscriptional regulation of T-cadherin in human osteosarcoma cells [41]. The role of sex hormones in the phenotypic effect of* CDH13* polymorphisms required further research.

*Limitations*. The main limitation of our study was its modest sample size, which was not analyzed in any functional manner and showed only an arguable relationship with the phenotypes. The replication of our results using a second cohort, particularly by one with a larger sample size and a different ethnic population, would strengthen the validity of our analysis. In addition, the cross-sectional nature of the present study limits our ability to infer a causal relation between* CDH13* variants, inflammatory marker levels, and various immune-mediated and inflammatory disorders.

## 5. Conclusion

Our data suggested multiple mechanisms involved in the association between adiponectin and inflammatory marker levels, in which T-cadherin plays a crucial role in circulating adhesion molecule levels.* CDH13* genotypes with low adiponectin levels were found to be associated with a more favorable metabolic profile but a higher risk profile regarding inflammatory marker levels. These results, when combined with previous studies, may provide a fuller explanation of the diverse effects of adiponectin.

## Figures and Tables

**Table 1:** Baseline characteristics of the health of study participants.

	Total	Men	Women	*P* value
Number	530	270	260	
Age (years)	44.9 ± 9.4	43.9 ± 9.3	45.9 ± 9.3	0.013
Body mass index (kg/m^2^)	24.2 ± 3.5	24.8 ± 3.2	23.5 ± 3.7	2.2 × 10^−5^
Waist circumference (cm)	84.6 ± 9.7	87.4 ± 7.8	81.61 ± 10.5	1.6 × 10^−12^
Hypertension	9.6%	8.9%	10.4%	0.331
Diabetes mellitus	2.5%	2.6%	2.3%	0.528
Obesity	36.6%	44.1%	28.8%	1.9 × 10^−4^
Adiponectin (mg/L)	7.25 ± 4.90	5.45 ± 3.44	9.13 ± 5.45	7.8 × 10^−21^
CRP (mg/L)	1.04 ± 1.35	1.09 ± 1.40	0.99 ± 1.31	0.060
Fibrinogen (mg/dL)	262.6 ± 68.9	260.3 ± 70.9	265.0 ± 66.9	0.432
sE-selectin (ng/mL)	52.7 ± 25.6	60.0 ± 26.8	45.0 ± 21.8	4.1 × 10^−14^
sP-selectin (ng/mL)	136.9 ± 115.0	149.8 ± 128.9	123.6 ± 97.0	0.004
sVCAM1 (ng/mL)	488.1 ± 132.7	491.4 ± 153.0	484.86 ± 108.0	0.724
sICAM1 (ng/mL)	241.0 ± 112.8	245.6 ± 109.7	236.1 ± 116.0	0.215
sTNFR2 (pg/mL)	3240.4 ± 922.4	3308.8 ± 969.1	3169.5 ± 867.7	0.082
MCP1 (pg/mL)	73.5 ± 60.6	80.0 ± 70.7	66.8 ± 47.1	0.011
IL6 (pg/L)	4.1 ± 7.6	4.4 ± 9.0	3.9 ± 5.8	0.569
MMP1 (pg/mL)	483.8 ± 1201.4	351.9 ± 585.6	620.2 ± 1597.2	0.850
MMP2 (ng/mL)	126.0 ± 40.9	122.4 ± 41.8	129.8 ± 39.6	0.038
MMP9 (ng/mL)	143.7 ± 112.5	156.7 ± 114.4	130.3 ± 109.2	0.008
SAA (mg/L)	5.90 ± 15.42	6.68 ± 19.64	5.10 ± 9.32	0.374

CRP: C-reactive protein; sE-selectin: soluble E-selectin; sP-selectin: soluble P-selectin; sVCAM1: soluble vascular cell adhesion molecule-1; sICAM1: soluble intercellular adhesion molecule-1; TNFR2: tumor necrosis factor-alpha receptor 2;  MCP1: monocyte chemotactic protein-1; IL6: interleukin-6; MMP1: matrix metalloproteinase-1; MMP2: matrix metalloproteinase-2; MMP9: matrix metalloproteinase-9; SAA: serum amyloid A. Continuous variables are presented as mean ± SD.

CRP: patients with CRP levels >10 mg/L excluded.

**Table 2:** Associations between adiponectin levels and adiposity status and inflammatory marker levels in Han Chinese patients in Taiwan.

Clinical and biochemical parameters		Unadjusted		Adjusted for age, sex, BMI, and smoking status	
		*r*	*P* value	*r*	*P* value
Anthropology	Age (years)	0.164	1.50 × 10^−4^		
	Body mass index (kg/m^2^)	−0.356	2.97 × 10^−17^	−0.321	4.18 × 10^−14^*∗*^^
	Waist circumference (cm)	−0.365	3.84 × 10^−18^	−0.297	3.25 × 10^−12^*∗*^^

Inflammation markers	CRP (mg/L)	−0.272	2.87 × 10^−10^	−0.186	2.22 × 10^−5^
	Fibrinogen (mg/dL)	−0.057	0.189	−0.055	0.210
	sE-selectin (ng/mL)	−0.318	9.77 × 10^−14^	−0.150	0.001
	sP-selectin (ng/mL)	−0.119	0.006	−0.073	0.096
	sVCAM1 (ng/mL)	0.163	1.72 × 10^−4^	0.142	0.001
	sICAM1 (ng/mL)	−0.134	0.002	−0.114	0.009
	sTNFR2 (pg/mL)	−0.045	0.298	−0.004	0.923
	MCP1 (pg/mL)	−0.050	0.252	−0.003	0.948
	IL6 (pg/L)	−0.065	0.143	−0.033	0.463
	MMP1 (pg/mL)	−0.007	0.878	−0.016	0.719
	MMP2 (ng/mL)	0.119	0.006	0.062	0.159
	MMP9 (ng/mL)	−0.113	0.010	−0.043	0.330
	SAA (mg/L)	−0.009	0.839	0.041	0.352

Abbreviations as in Table 1.

CRP: patients with CRP levels >10 mg/L excluded.

^*∗*^Adjusted for age, sex, and smoking status only.

**Table 3:** Adiponectin levels: stepwise linear regression analysis.

Variable	*R* ^2^ ^a^	Beta	*P* value
Sex	0.152	0.163	7.9 × 10^−13^
Body mass index (kg/m^2^)	0.232	−0.016	1.71 × 10^−6^
sVCAM1 (ng/mL)	0.253	0.566	7.49 × 10^−7^
sE-selectin (ng/mL)	0.282	−0.180	0.003
sICAM1 (ng/mL)	0.295	−0.189	0.003
Age (per year)	0.307	0.004	0.001
CRP (mg/L)	0.319	−0.066	0.002

^a^Cumulative *R* ^2^. Multiple linear regression, adjusted for age, gender, and smoking status.

**Table 4:** Association between *CDH13 *rs12051272 genotypes and adiposity status and adiponectin levels and inflammatory marker levels.

Genotype		GG (*N* = 235)	GT (*N* = 229)	TT (*N* = 57)	*P*1	*P*2	*P*3	Β (95% CI)^*∗*^	*P*1^*∗*^	*P*2^*∗*^	*P*3^*∗*^
Anthropology	Age (years)	44.6 ± 8.2	45.3 ± 10.5	44.8 ± 9.3							
	Body mass index (kg/m^2^)	23.7 ± 3.0	24.4 ± 3.8	25.0 ± 3.8	0.012			1.07 (0.12–2.02)	0.027		
	Waist circumference (cm)	83.5 ± 8.8	84.8 ± 10.0	88.3 ± 11.0	1.31 × 10^−4^	0.004	0.011	4.68 (2.17–7.19)	2.71 × 10^−4^	0.003	0.008
	Adiponectin (mg/L)	8.58 ± 5.44	6.40 ± 3.98	5.15 ± 4.38	2.7 × 10^−10^	7.45 × 10^−9^		−0.20 (−0.27 to −0.13)	2.57 × 10^−8^	3.52 × 10^−7^	

Inflammation marker	CRP (mg/L)	1.10 ± 1.51	0.88 ± 1.06	1.44 ± 1.60	0.018	0.163	0.702	0.18 (0.04–0.31)	0.010	0.084	0.395
	Fibrinogen (mg/dL)	261.7 ± 71.6	261.4 ± 62.8	273.2 ± 79.7	0.241	0.463	0.646	11.59 (−7.19–30.38)	0.226	0.412	0.563
	sE-selectin (ng/mL)	52.8 ± 21.7	49.76 ± 20.70	65.08 ± 47.16	0.018	0.083	0.327	0.07 (0.02–0.12)	0.009	0.039	0.160
	sP-selectin (ng/mL)	137.6 ± 104.8	132.4 ± 119.7	165.0 ± 137.7	0.093	0.116	0.230	0.08 (−0.0004–0.16)	0.051	0.064	0.126
	sVCAM1 (ng/mL)	485.9 ± 107.4	479.2 ± 105.5	537.6 ± 263.7	0.027	0.014	0.001	0.03 (0.01–0.06)	0.019	0.010	0.001
	sICAM1 (ng/mL)	236.8 ± 95.2	235.3 ± 89.8	286.1 ± 16.00	0.024	0.058	0.171	0.06 (0.01–0.11)	0.016	0.037	0.103
	sTNFR2 (pg/mL)	3222.1 ± 900.7	3230.7 ± 939.5	3411.4 ± 964.8	0.125	0.194	0.193	0.03 (−0.01–0.06)	0.121	0.180	0.180
	MCP1 (pg/mL)	70.8 ± 61.6	79.1 ± 65.1	65.0 ± 33.2	0.914	0.845	0.844	−0.02 (−0.10–0.06)	0.618	0.564	0.558
	IL6 (pg/L)	4.7 ± 10.0	3.4 ± 4.7	4.8 ± 5.9	0.065	0.129	0.165	0.12 (0.002–0.0.24)	0.046	0.221	0.107
	MMP1 (pg/mL)	518.1 ± 1341.1	472.0 ± 1174.5	446.2 ± 675.8	0.221	0.232	0.240	0.09 (−0.04–0.22)	0.186	0.197	0.204
	MMP2 (ng/mL)	124.4 ± 35.4	127.6 ± 44.7	123.8 ± 46.0	0.749	0.863	0.877	−0.02 (−0.05–0.02)	0.356	0.437	0.748
	MMP9 (ng/mL)	145.9 ± 122.3	139.2 ± 97.6	156.5 ± 131.0	0.359	0.504	0.685	16.37 (−15.44–48.19)	0.313	0.429	0.574
	SAA (mg/L)	6.32 ± 13.92	5.35 ± 17.77	6.35 ± 11.80	0.618	0.865	0.597	0.05 (−0.09–0.19)	0.462	0.653	0.439

Abbreviations as in Table 1.

*N* = number; CRP: excluded subjects with CRP levels >10 mg/L;  *P*1 value adjusted for age and sex; *P*2 value adjusted for age, sex, BMI, and smoking status; *P*3 value further adjusted for adiponectin levels; sICAM1: only subjects with *ICAM1* rs5491 AA genotype selected; ^*∗*^data was analyzed in recessive model (GG + GTversus TT).

## Abbreviations

GWAS: Genome-wide association studies
BP: Blood pressure
BMI: Body mass index
ELISA: Enzyme-linked immunosorbent assay
CRP: C-reactive protein
SAA: Serum amyloid A
sICAM1: Soluble intercellular adhesion molecule-1
sVCAM1: Soluble vascular cell adhesion molecule-1
sE-selectin: Soluble E-selectin
MMP9: Matrix metalloproteinase-9
MCP1: Monocyte chemotactic protein-1
IL6: Interleukin-6
MMP2: Matrix metalloproteinase-2
MMP1: Matrix metalloproteinase-1
sP-selectin: Soluble P-selectin
sTNFRII: Soluble tumor necrosis factor receptor 2.
